# Hints on Pelvic Diseases of Women

**Published:** 1901-08

**Authors:** C. E. Boynton


*Editor Atlanta Journal-Record of Medicine:*

### HINTS ON PELVIC DISEASES OF WOMEN.

*Editor Atlanta Journal-Record of Medicine:*

1. In one locality where I practiced for three years I found *no gonorrhea* and much pelvic disease.

2. In another locality I found plenty of both.

3. My conclusion is that gonorrhea may cause possibly fifteen per cent. of the pelvic diseases of women, but not more.

4. Constipation after confinement, leading up to autointoxication, *is one* cause of female pelvic trouble.

5. Constipation (*i. e.,* a loaded bowel) during confinement is another cause.

6. Neglected anemia, over lactation, hard work, poor food, bad teeth, bad sexual mating—all predispose to pelvic disease.

7. I have known a lively attack of gonorrhea to improve an irritable female urethra and leave a woman in better health afterwards, with positively no discharge after a few weeks and very little treatment, cure being nearly spontaneous.

8. In ten years of country practice I have seen four or five cases of syphilis, yet in city practice I found syphilis quite frequent.

9. High altitude (*i. e.,* 4,000 to 10,000 feet above sea level) ages women, and predisposes also to pelvic disease.

10. I have practiced in seaport villages where pelvic disease was rare, while abortion (criminal) and gonorrhea were plenty enough.

11. An inland district, remote from markets, often proves unhealthful, simply because people do not get enough to eat. Nothing to cook makes poor cooks, and poor cooks suffer more frequently from pelvic diseases.

12. Where fish is cheap and plentiful primitive culinary arts may suffice.

C. E. BOYNTON, M.D.

